# PRECISE pregnancy cohort: challenges and strategies in setting up a biorepository in sub-Saharan Africa

**DOI:** 10.1186/s12978-020-0874-7

**Published:** 2020-04-30

**Authors:** Rachel Craik, Donna Russell, Rachel M. Tribe, Lucilla Poston, Geoffrey Omuse, Patricia Okiro, David Chege, Mathurin Diatta, Abdul Karim Sesay, Inocencia Cuamba, Carla Carrilho, Esperança Sevene, Meriel Flint-O’Kane, Peter von Dadelszen, The PRECISE Network, Umberto D’Alessandro, Anna Roca, Hawanatu Jah, Ofordile Ogochukwu, Andrew Prentice, Melisa Martinez-Alvarez, Brahima Diallo, Adbul Sesay, Kodou Lette, Alpha Bah, Chilel Sanyang, Marleen Temmerman, Angela Koech Etyang, Peris Musitia, Mary Amondi, David Chege, Patricia Okiro, Geoffrey Omuse, Sikolia Wanyonyi, Esperança Sevene, Paulo Chin, Corssino Tchavana, Salesio Macuacua, Anifa Vala, Helena Boene, Lazaro Quimice, Sonia Maculuve, Eusebio Macete, Inacio Mandomando, Carla Carillho, Peter von Dadelszen, Laura A. Magee, Meriel Flint-O’Kane, Rachel Craik, Amber Strang, Marina Daniele, Donna Russell, Prestige Tatenda Makanga, Liberty Makacha, Yolisa Prudence Dube, Newton Nyapwere, Lucilla Poston, Jane Sandall, Rachel M. Tribe, Andrew Shennan, Sophie Moore, Tatiana Salisbury, B M Barett, Lucy Chappell, Sean Beevers, Kate Bramham, Aris Papageorgiou, Alison Noble, Hannah Blencowe, Veronique Filippi, Joy Lawn, Matt J Silver, Matthew Chico, Judith Cartwright, Guy Whitley, Sanjeev Krishna, Marianne Vidler, Jing ( Larry) Li, Jeffrey Bone, Mai-Lei ( Maggie) Woo Kinshella, Beth A. Payne, Domena Tu, Warancha Tumtaweetikul, William Stones

**Affiliations:** 1 Department of Women and Children’s Health, School of Life Course Sciences, King’s College London, London, UK; 2 Nuffield Department of Women’s and Reproductive Health, University of Oxford, Oxford, UK; 3 Donna Russell Consultants, Seattle, USA; 4 Department of Pathology, Aga Khan University Hospital, Nairobi, Kenya; 5 Medical Research Council Unit (The Gambia) at the London School of Hygiene and Tropical Medicine, Fajara, The Gambia; 6 Centro de Investigacão em Saúde de Manhiça, Maputo, Mozambique; 7 Department of Pathology, Maputo Central Hospital, Maputo, Mozambique; 8 Department of Physiological Science, Clinical - Pharmacology, Faculty of Medicine, Universidade Eduardo Mondlane, Maputo, Mozambique; 9 Faculty of Public Health and Policy, London School of Hygiene and Tropical Medicine, London, UK

**Keywords:** Biorepository, PRECISE, Pre-eclampsia, Preterm birth, Hypertension, Pregnancy, Biological specimens

## Abstract

**Background and objective:**

PRECISE is a population-based, prospective pregnancy cohort study designed for deep phenotyping of pregnancies in women with placenta-related disorders, and in healthy controls. The PRECISE Network is recruiting ~ 10,000 pregnant women in three countries (The Gambia, Kenya, and Mozambique) representing sub-Saharan Africa. The principal aim is to improve our understanding of pre-eclampsia, fetal growth restriction and stillbirth. This involves the creation of a highly curated biorepository for state of the art discovery science and a rich database of antenatal variables and maternal and neonatal outcomes. Our overarching aim is to provide large sample numbers with adequate power to address key scientific questions. Here we describe our experience of establishing a biorepository in the PRECISE Network and review the issues and challenges surrounding set-up, management and scientific use.

**Methods:**

The feasibility of collecting and processing each sample type was assessed in each setting and plans made for establishing the necessary infrastructure. Quality control (QC) protocols were established to ensure that biological samples are ‘fit-for-purpose'. The management structures required for standardised sample collection and processing were developed. This included the need for transport of samples between participating countries and to external academic/commercial institutions.

**Results:**

Numerous practical challenges were encountered in setting up the infrastructure including facilities, staffing, training, cultural barriers, procurement, shipping and sample storage. Whilst delaying the project, these were overcome by establishing good communication with the sites, training workshops and constant engagement with the necessary commercial suppliers. A Project Executive Committee and Biology Working Group together defined the biospecimens required to answer the research questions paying particular attention to harmonisation of protocols with other cohorts so as to enable cross-biorepository collaboration. Governance structures implemented include a Data and Sample Committee to ensure biospecimens and data will be used according to consent, and prioritisation by scientific excellence. A coordinated sample and data transfer agreement will prevent delay in sample sharing.

**Discussion:**

With adequate training and infrastructure, it is possible to establish high quality sample collections to facilitate research programmes such as the PRECISE Network in sub-Saharan Africa. These preparations are pre-requisites for effective execution of a biomarker-based approach to better understand the complexities of placental disease in these settings, and others.

## Introduction

### The PRECISE network cohort study

The PRECISE (PREgnancy Care Integrating translational Science Everywhere) Network has enabled a population-based, prospective cohort study designed to improve our understanding of the mechanisms leading towards placental disease and related pregnancy complications in sub-Saharan Africa [1]. The cohort study aims to recruit a total of up to 10,000 pregnant women and, for comparison, 1800 non-pregnant women of reproductive age across the three countries: The Gambia, Kenya and Mozambique. Biological specimens are being collected longitudinally across the continuum of pregnancy, delivery, and the neonatal period. In addition, sites are consenting women who present at the health facility with complications during pregnancy. All sites will collect a comprehensive set of associated phenotypic information to deeply characterise these pregnancies [2]. Table 1 describes the cohorts with expected recruitment per country.

**Table 1:** Recruitment by country and cohort

Country	Facility	Non-pregnant women of reproductive age	Unselected Pregnant Women	Pregnant Women Presenting with Complications	Total Women Recruited
The Gambia:					
Farafenni	District Hospital	600	2000	300	2900
Illiasa,	Rural PHC				
Ngayen Sanjal	Rural PHC				
Kenya:					
Mariakani	Subcounty Hospital	600	4000	600	5200
Rabai	Rural Health Centre				
Mozambique:					
Manhica	Tertiary Referral Centre	600	4000	600	5200
Xinavane	Rural Hospital				
Total		1800	10,000	1500	13,300

The samples will establish a biorepository which will be accessible to the PRECISE Network for research to identify biological markers (e.g., genomic, metagenomic, proteomic, metabolomic, hormonal and inflammatory) as predictors of important maternal and fetal outcomes. Samples remaining after the initial analysis will be available to other approved researchers to facilitate and accelerate future discoveries on maternal, fetal, and neonatal health. Another major objective of this study is to build individual and institutional research capacity across Africa. The biorepositories will be a significant resource for all of Africa [3], enabling ongoing multi-omics research on pregnancy complications as well as the developmental origins of health and disease.

Here we describe the methodology applied to setting up the infrastructure and governance for the development of this large biorepository in these sub-Saharan African countries.

## Methods

### Optimal sample collection

To carry out scientific research at the highest standard, collection and curation of the biorepository has to be optimised to ensure quality. This requires rigorous protocols for handling of samples and storage to avoid biological degradation according to evidence-based best practice [4]. The PRECISE team, with assistance from an experienced consultant in this field, adhered to International Society for Biological and Environmental Repositories (ISBER), NIH, and NCI Best Practices for establishing and operating biorepositories [5, 6]. This included an initial assessment of biobanking capabilities, including but not limited to, laboratory and storage facilities, IT, staffing, security, and emergency/disaster plan. PRECISE Standard operating procedures (SOPs) were developed for each sample type from collection to final storage and labelling. The PRECISE SOPs are harmonised with other pregnancy cohort sample collections across (low- and middle-income countries (LMICs) so that samples from multiple sites are comparable and can be pooled for large-scale studies. The SOPs were translated to the appropriate languages for the sites and included protocols to control pre-analytical variables to protect protein, DNA and RNA integrity, including**:**

- Avoidance of haemolysis during venepuncture of blood samples
- Time stamping to document length of time sample is at room temperature: before processing, and between processing and placing into storage
- Documenting visual quality of blood derivatives (e.g., colour of plasma)
- Continuous monitoring of temperatures of sample storage units (freezers and liquid nitrogen tanks) to assure constant optimum temperature
- Documenting calibration of equipment used for processing and analysing samples

### Quality control

A procedure has been established to routinely assess sample quality. The primary method that PREICSE is following for quality control is ensuring strict adherence to the protocol and SOPs. This is accomplished by extensive training and frequent site visits at the start of collections to monitor collections and processing as well as safety. Additionally, the PRECISE coordinator schedules weekly calls with the teams to address any challenges and to share experiences and learnings.

Best Practices recommends assessing quality of specimens throughout the collection period. As testing quality means using up valuable resources for research, it was decided to test approximately 2 % of samples from the bioresource at regular intervals across the length of the project for quality control checks. The sampling protocol ensures the numbers tested are adequate to confirm quality but at the same time avoiding depletion of the bioresource. Sampling is structured to include participants across all sites for the entire duration of the project.

The PRECISE Network Biorepository Quality Assessment Protocol includes assessment of DNA and RNA purity and concentration as well as RNA integrity in blood and tissue samples. For placental histology, PRECISE will use standard haematoxylin- and eosin- (H&E) stained slides, as well as at least one immunohistochemistry marker to check on the viability of the tissues for antigen retrieval. Table 2 indicates the cut-offs for the quality standards.

**Table 2:** Quality Measures for sample RNA, DNA and histology. RIN (RNA Integrity Number), DIN (DNA Integrity Number), H&E haematoxylin- and eosin-staining, IHC Immuno-histochemistry

Specimen type	Primary	Secondary
RNA	RIN value ≥7.0	260/280 ratio: 1.9–2.1
DNA	DIN value ≥7.0	260/280 ratio: 1.7–1.9
Placenta	RIN value ≥7.0	RNA Extraction 260/280 ratio
Placenta Histology	H&E	IHC Markers e.g. vimentin

## Overview of bioresource protocol

The PRECISE Biology Working Group defined the biospecimen collection framework (Table 3) and the sample collection protocol (Table 4). Phenotypic data will be collected at each visit where biospecimens are taken [2].

**Table 3:** Overview of type of biological samples & the collection schedule

Time of sample collection	Type of biological sample
PRECISE visit 1	Maternal blood, urine, mid-vaginal swabs
PRECISE visit 2 (≥28^+ 0^ weeks)	Maternal blood and urine
Intrapartum (at the onset of labour)	Maternal mid-vaginal swab
Delivery	Umbilical cord blood, placenta and cord tissue, placental membranes
Within 48 h post-partum	Newborn heel prick only if cord blood is not collected (only The Gambia and Mozambique), maternal blood and urine.
Post-partum visit (6 weeks after delivery)	Neonatal stool sample, maternal blood and urine. Neonatal heel prick (if required, Kenya only)

**Table 4:** Summary of sample types collected in PRECISE

Sample type	Collection tube	Aliquots	Storage
Blood	EDTA	Blood spot	Room temperature
	EDTA	Whole blood	−80 °C
	EDTA	Buffy coat	−80 °C
	EDTA	Plasma	−80 °C
	Serum	Serum	−80 °C
Urine	no treatment	Urine	−80 °C
		Urine sediment	−80 °C
Vaginal swab	Copan eSwab	TE buffer	−80 °C
	Dacron swab	Protease inhibitor	−80 °C
Placenta, fetal membranes and cord	No treatment	Peripheral biopsies	Snap frozen
		Central biopsies	Snap frozen
		Peripheral biopsies	Paraffin embedding
		Central biopsies	Paraffin embedding
		Cord tissue	Snap frozen
		Cord tissue	Paraffin embedding
		Membrane	Paraffin embedding
Heel prick	No treatment	Blood spot	Room temperature
Neonatal stool	DNA/RNA Shield tube	Swab	−80 °C

### Biospecimen collection and processing

*Maternal and cord blood*: A blood spot and aliquots of whole blood, plasma, buffy coat will be extracted from blood collected in an EDTA vacutainer. Serum aliquots will be obtained from blood collected in a siliconized vacutainer.

*Maternal urine*: Aliquots of untreated urine and urine sediment will be stored.

*Mid-vaginal swabs*: Two swabs for DNA extraction and sequencing will be immediately placed in Tris-EDTA (TE) buffer for storage and two additional swabs will be collected in PBS plus protease inhibitor for biochemical analysis.

**Fig. 1:**
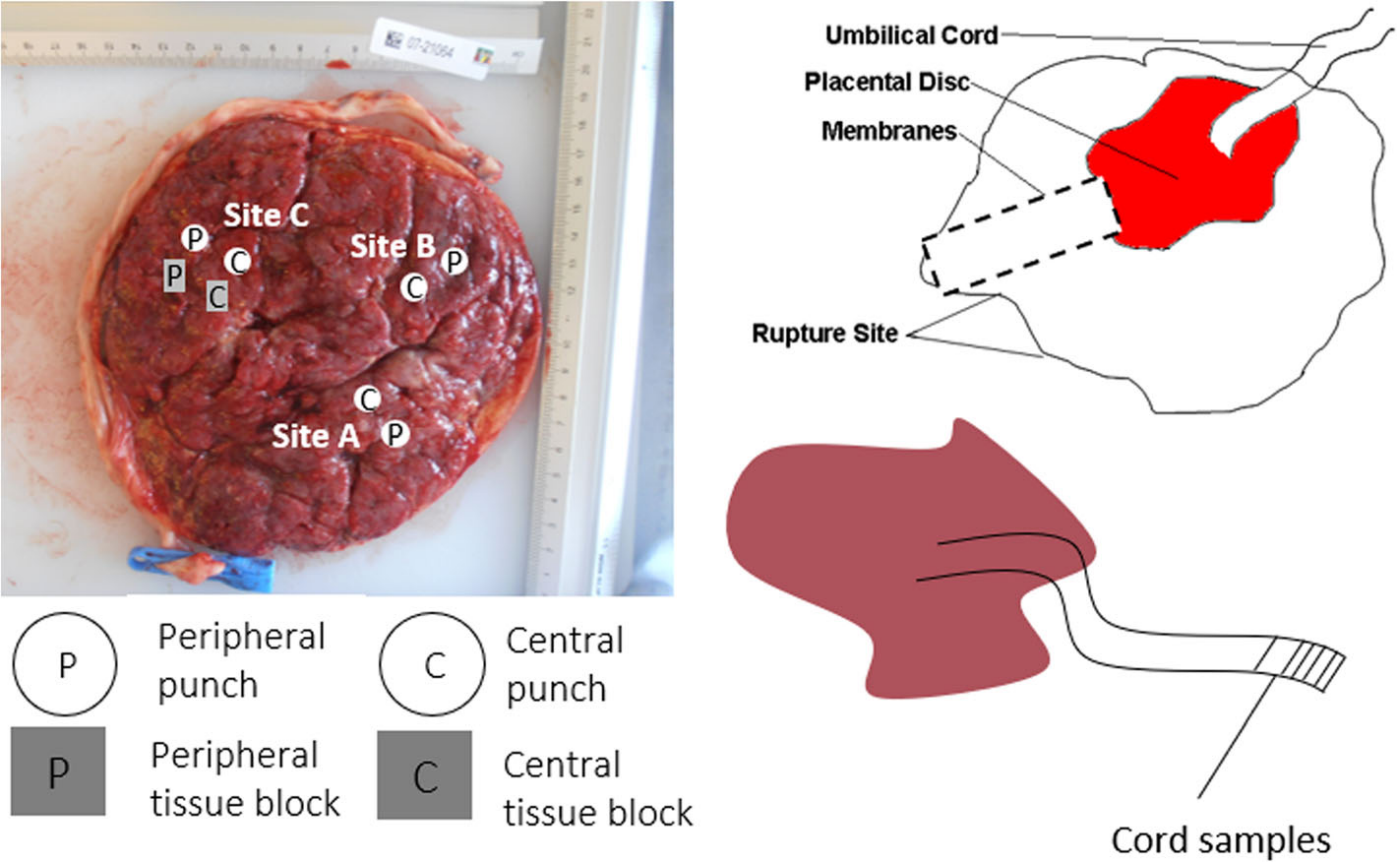
Placenta processing protocol – the figure illustrates where the samples from the placenta tissue, membranes and cord are collected

*Placental, cord and membrane tissue samples:* These samples will be collected and processed within 30 min of delivery (Fig. 1 demonstrates placenta protocol). Full thickness central and peripheral sampling of the placenta will be undertaken and samples snap frozen in LN_2_. Corresponding samples will be stored in formalin for histology. Cord tissue samples will be snap frozen in LN_2_ and another sample placed in formalin. A fetal membrane sample will retained in formalin for histology. Photographs and trimmed weight are also acquired and stored.

*Delayed cord blood:* In cases where the cord blood is not available immediately, a sample of the clotted cord blood will be taken for provision of newborn DNA only.

*Newborn heel prick:* In the absence of cord blood, a newborn heel prick blood sample will be taken for a DNA sample or taken at the 6 week follow-up visit.

Whilst the protocol dictates that specimens are promptly processed before being placed into aliquots and short-term storage, practicalities necessitated minor procedural differences between countries in order to achieve this. Therefore, in The Gambia and Mozambique, aliquots will be temporarily stored in LN_2_ (gas phase) until transported to the main bioresource facility where they are transferred to − 80 °C freezers for long-term storage. In Kenya, samples will be stored immediately in − 80 °C freezers once processed.

### Biorepository governance

Approval for the study, including sample collection and storage, was obtained from all participating institutions, hospitals and relevant ethics committees. The principle of sharing samples between sites was included in the ethics approval which has been granted in each of the participating countries. The biorepository samples belong to the country of origin and will not be collated into a central biobank.

A Data and Sample Committee (DSC) has been convened to act as the governing authority of the PRECISE biorepository and to provide guidance and oversight for the collection, processing and utilisation of the samples.

The committee is composed of 13 members, six core members defined by their role in the study and seven subject matter experts. The core members are the PRECISE Chief Investigator, the Principal Investigator from each site, chair of the Biospecimen Quality Committee, and Biorepository Research Co-ordinator (Secretary). The chair can appoint up to seven knowledgeable experts and community representatives with special qualifications and competence to deal effectively with specific biorepository or data issues.

The DSC follows a clearly outlined mandate which includes:

- Providing oversight and assurance of the quality of samples being collected across three sites. This will involve reviewing the regular reports from the Biospecimen Quality Committee and providing written feedback. This may action site visits or additional quality control measures.
- Controlling access to the PRECISE samples and data including reviewing applications for samples. The PRECISE Study Sample Request Form will be provided to all interested parties. The form requires details of the scientific rationale, justification of the requested sample numbers and volume (including power calculations), data fields required from the PRECISE data dictionary and a data analysis plan. The form requires one of the PRECISE PIs to be a co-applicant on the proposed study. The proposed study will be reviewed for scientific merit, scientific priority according to the PRECISE aims, overlap with previously agreed studies and for sample availability.
- Ensuring samples and data are shared in with the consent received and the data and sample transfer agreements arranged between institutions. This includes forming a collaborative group of the technology transfer officers from each of the countries to facilitate sample and data transfer and avoid unnecessary delay.
- Establishing policies for the long-term sustainability and utilisation of the biorepository.

## Results

### Barriers encountered in developing the biorepository

Several challenges were overcome whilst setting up the biorepository. After site visits from the team to scope out the available resources and facilities, several issues and considerations were identified. These are described below.

#### Capacity building

*Facilities:* As anticipated, nearly all the rural sites required renovations to their facilities prior to study commencement. In The Gambia, the new field sites required significant work to renovate existing facilities including an external storage room for the liquid nitrogen equipment in the main hospital, building a laboratory in one of the rural clinics and refurbishing the laboratory in the second rural clinic. In Kenya, in the rural clinic, the laboratory had to be extended to provide the required space. In Mozambique, the rural site also required renovation work on the laboratory. This incurred significant costs, some unanticipated due to locally poor infrastructure. This has benefited sites greatly as these improvements to the laboratory infrastructure will inevitably contribute to improved patient care as well as enabling a new population to become engaged in research.

*Staffing:* Co-ordinating the timing of staff recruitment to a multi-centre study of this size was challenging. In some sites we faced delays in hiring key staff (e.g., data managers and laboratory technicians) primarily because staff were needed for specialised roles with a limited pool of suitable candidates. Most of the staff were recruited directly to the study with a minority being seconded from an existing post. In some instances, budget allocations had to be exceeded in order to recruit staff with the requisite qualifications and training. Staff turnover resulted in additional challenges before the launch; including site co-ordinator, laboratory manager and data manager posts. However, with additional support and training, all teams were in place by the start date to ensure smooth delivery of the study.

*Training:* None of the field sites participating in the study had experience of such an extensive collection of biospecimens. The field staff required training in sample collection, processing, and storage and the PRECISE Baobab laboratory information management system (LIMS), (https://baobablims.org/). As many of the staff were not experienced in working in laboratories, all staff had to complete good clinical practice (GCP) and good clinical laboratory practice (GCLP) courses and liquid nitrogen training to ensure safety in the laboratory. This training was facilitated by The Global Health Network (https://tghn.org), which hosts short courses online in clinical research to facilitate high-quality research. We used the train-the-trainer model, where in a central training session held in The Gambia, the laboratory leads from each site were trained in sample collection and processing. These staff then trained all the staff in their site in their local language where the teams had access to the SOPs, instructions and forms in their local language. The biobanking lead visited each site prior to commencement of the study and will visit again within 6 months following the start of recruitment to provide any necessary further training and solve any issues. Reinforcement training will be delivered via online webinars, online conferencing and further site visits.

The PRECISE PIs are also committed to capacity building to enable training of scientists and bioinformaticians in the African countries to enable the acquisition of the necessary skill base to undertake local analysis of the biorepository samples. Whilst not included in the original funding application, ongoing funding applications include provision for these activities.

*Cultural barriers:* Community engagement is critical to gaining local acceptance and enthusiasm for the project. This has started in each site through meetings with local community leaders, healthcare professionals, religious leaders and institutional leaders as well as meetings with women and their families in each local community. From the work done so far, we have found, for example, that there is resistance to blood sampling in pregnancy over and above that needed for routine clinical care, as it is perceived too much blood is being collected. This issue has been raised by field workers and clinical staff as well as by women being recruited, and other community members. To overcome this, extensive training of the field workers and nurses was undertaken to educate and inform them that the volumes required are not harmful. This training will continue throughout the study. The collection of the placenta was also a new concept to many of these communities, especially where women take the placenta home after delivery. Again, this concern was, and will continue to addressed through education and community engagement. In one of the countries, taking blood from the newborn before leaving the facility was considered to be stigmatising to the family as this is typically limited to the infants of HIV-positive women. For that reason, the protocol was modified so that in cases where the cord blood collection was either refused or missed, a heel prick will be performed at the 6 week postpartum visit. The breastfeeding questions had to be well phrased to avoid any HIV implications. HIV-positive mothers are sensitive to being asked if they are breastfeeding due to perceptions linking bottle feeding with HIV-positive status. Extensive training was done to ensure staff are sensitive in their approach and questions are so phrased that women did not feel stigmatised.

*Procurement:* In recognition of purchasing power of bulk buying, most consumables (excluding liquids) and equipment were ordered by the central co-ordinating team and shipped to the sites. Whilst this ensured consistency in materials, despite suppliers’ intentions, shipping was often delayed mainly due to stock issues, especially for custom-made items (freezer racking, liquid nitrogen generators) or where large orders were placed (blood spot cards).

*Shipments and taxes:* Discussions were held with all sites prior to commencement of the study regarding importation of essential laboratory consumables and equipment and the necessary procedures included in the study planning. However, some local and unanticipated delays were incurred. In Kenya, for example, recently implemented importation regulations requires an inspection of goods being sent to obtain a shipping permit before leaving the country of origin. Introduced recently, a permit from the Pharmacy and Poisons Board and the Energy Regulatory Commission was needed for -80 °C freezers. As this process was not yet streamlined, this led to a delay of 6 months for freezer importation. Although anticipated, import taxes in Mozambique were ≥ 50% of the value of the goods but, once paid, the importation process was relatively straightforward.

*Baobab LIMS customisation:* The PRECISE biorepositories use the web-based Baobab LIMS sample tracking. The Baobab LIMS was developed by African and European researchers as an open-source LIMS to help harmonise biobanks across Africa. Each country hosts their own database on their servers ensuring each site only has access to their own data. Access to the database is restricted by username and password. Establishing the database, programming the database and staff training incurred some minor delays. Despite this, the PRECISE team remain very supportive of an open-source sample solution resource for Africa.

*Sample storage:* Several of the sites do not have a reliable electricity connection making − 80 °C freezers less attractive. As an alternative, liquid nitrogen (LN_2_) was considered; however dependable sources were not always available. To circumvent these issues liquid nitrogen generators were purchased for sites in The Gambia and Mozambique. Samples are stored in LN_2_ tanks temporarily in the field before being moved to − 80 °C freezers at the main biobanking facilities, which have reliable electricity. For Kenya, where freezers were installed, freezer alarm systems were put in place. Due to paucity of reliable in-country biorepositories, one of the challenges faced was finding suitable off site back up biorepositories to enable split sample storage or transfer of samples in the event the primary storage failed. Rather, each site has a stringent plan in the event that a sample storage unit fails: this includes back-up power supplies for freezers and spare storage space in alternative units. For example, in the event of a power failure, all freezers have a site specific alarm system including mobile phone alerts.

### Work flow processes

A number of processes were built into the PRECISE work flow to ensure quality samples are obtained and to minimise any errors. These include:

- *Participant ID numbers:* Each participant identification number is unique and contains a ‘check digit’ to detect any errors in data entry. This check digit will detect any single digit errors or any adjacent transposition errors. This check digit, in combination with the ID being in a 2D barcode which can be scanned, should minimise the risk of any data or samples being linked to the wrong participant**.** These ID numbers are generated by the central data management team and are used to link the clinical data to the biological sample data which are stored in separate databases.
- *Kit building:* To help with the sample collection process, sample collection kits are prepared by each site. These kits all have a unique kit number and contain all the required consumables for sample collection and processing. The sample aliquots are assigned a unique sample identification number which is linked to the kit ID number. The sample IDs are printed on the sample storage tubes using thermal printing technology. The printed text is resistant to alcohol, water, liquid nitrogen and mechanical abrasion and is stable over a wide temperature range making it durable. During the kit building process, the final step, for QC purposes, is to weigh the kit. If the weight is not as expected, the kit contents can be checked to ensure all the required consumables are present.

## Discussion

Biobanking is becoming increasingly recognised and utilised in Africa in a variety of fields including stroke [7], oncology [8], infectious disease [9] and pregnancy. This summary represents a practical account of the necessary stages required in setting up a large multi-country biorepository and database in low income settings. Whilst confined to three sub-Saharan African countries, the issues covered will be translatable to other countries in similar settings and, it is hoped will serve as a useful guide for others contemplating a study of similar complexity.

To ensure that PRECISE sites may participate in future collaborations and consortia, specimen collection has been designed to be harmonised with other similar large pregnancy cohort studies such as AMANHI [10], INTERBIO-21st [11], GAPPS [12] and HeLTI [13]. The PRECISE Network is also aligned with H3Africa goals [3] by building on existing infrastructure and resources to develop a cohort of African scientists focussed on significant global health problems that disproportionately affect women and families in sub-Saharan Africa.

*Facilities and sample storage:* The need to build specific facilities to house the scientific equipment and renovate laboratories led to unanticipated costs and time to setting up the repository; therefore, we recommend that ample contingency funding is included in any future applications for similar studies.

*Staffing:* Whilst central research management are keen to appoint site staff and avoid delays in initiating recruitment, there is also the potential in having a full team in place too early due to other unforeseen infrastructure issues which can delay the commencement of the study. This can lead to unnecessary staff costs and insufficient work in the team in the set-up phase. As in accordance with best practice, we planned to phase recruitment by starting recruitment in each country sequentially. From our experience, a phased recruitment process of staff within each centre might also be considered, starting with the core team and expanding to the field teams once timelines are in place.

*Training*: From our experience, regular contact and training with sites is essential to ensure compliance with SOPs and to pre-empt local deviations. Engaging with the site teams regularly boosts morale and keeps the teams motivated; this is essential when asking teams to process multiple samples according to a strict SOP. The PRECISE Annual Meetings have been proven to be excellent team building exercises with very positive feedback and should be preserved as a fundamental element of large research projects.

*Cultural*: Overcoming the cultural barriers to setting up this study, a planned activity, proved to be a vitally important activity of the programme which is not always included in project design. It is known that support from the communities reduces the challenges to such a complex endeavour [14], therefore extensive engagement with all levels of the community has been, and will continue to be, done to help in breaking down these barriers. For example, the anxiety about the volume of blood being taken was dispelled in The Gambia by demonstration of the actual volume using teaspoons. Being aware of cultural issues and developing systems to work around them is a pre-requisite for ongoing community support.

*Procurement, Shipments and Taxes:* Retrospectively, we would recommend purchase of consumables goods readily available in country and confine external purchasing to equipment and consumables not available locally. However, it must be recognised that sophisticated storage equipment is often not accessible and time is well spent in reviewing local availability of equipment and supplies. Given our experience, we advise that adequate time is built in to engage site teams early in the procurement process to identify and navigate local regulations and challenges so that the receipt of goods into their countries may be streamlined as much as possible.

In conclusion, the PRECISE project presented challenges but has proven the feasibility of setting up a complex biorepository in associate with a detailed clinical database. Development of the project was greatly facilitated by the inclusion of a highly experienced multi-disciplinary team and the early development of structured management system devolved to specialised groups. Everything is now in place for the implementation of the biorepository and the governance of it use as a shared resource.

## Data Availability

Not applicable.
